# Additional Personals

**Published:** 1899-12

**Authors:** 


### ADDITIONAL PERSONALS.

DRS. E. C. W. O'BRIEN and E. T. Smith have been appointed by the Sister Superior and the Medical advisory board of the Buffalo Hospital of the Sisters of Charity, as consultants to that institution in cases of diseases of the nervous system.

